# Psychometric evaluation of the Ethical Caring Competency Scale in nursing

**DOI:** 10.1186/s12912-022-00886-2

**Published:** 2022-05-03

**Authors:** Harumi Katayama, Taeko Muramatsu, Yoshimi Aoki, Eri Nagashima

**Affiliations:** Department of Fundamental Nursing, Hamamatsu University School of Medicine 1-20-1 Handayama, Higashi-ku, Hamamatsu city, Shizuoka, 431-3192 Japan

**Keywords:** Ethical caring, Scale, Competency, Rubric format, Reliability and validity

## Abstract

**Background:**

An ethical competence list for nurses could guide educators and managers in the field of health care to both support the development of ethical conduct and improve the assessment of ethical competence in health care.

**Aim:**

This study aimed to verify the reliability and validity of the Ethical Caring Competency Scale (ECCS) and to obtain suggestions for its use as an evaluation form in rubric format among a sample of Japanese nurses.

**Research design:**

This research employed a descriptive and cross-sectional design.

**Participants and research context:**

A self-administered questionnaire was distributed to 1157 nurses working in two hospitals in Japan. The contents of the survey were demographic data, a draft of the ECCS consisting of 22 competencies from four core competencies, questions regarding experience in learning about medical/nursing ethics, and the Work Motivation Measurement Scale for Nurses. Three levels of difficulty for the 22 items were established using relative comparisons of the mean scores within the four core competencies. Three groups, namely, an expert group, a middle group, and a beginner group, were categorized according to the quartiles of the total ECCS score.

**Ethical considerations:**

This study was approved by the Clinical Research Ethics Committee of Hamamatsu University School of Medicine (Decision no. 18-267). The ethical principles of voluntary participation, anonymity, and confidentiality were considered.

**Findings:**

A total of 962 valid responses were analyzed. The ECCS scores for the three levels of difficulty were significantly different from each other. Stability was confirmed by the test-retest of the total ECCS scores (*r* = .900, *p* < .0001). The total ECCS scores for the three groups showed significant differences in all pairs. The Cronbach’s *α* coefficient ranged from .72 to .89 for each core competency, and internal consistency was confirmed.

**Conclusion:**

The reliability and validity of the ECCS as a scale were statistically verified, and we were able to obtain suggestions for its application as a form of evaluation in rubric format.

## Introduction

Nurses face various ethical problems due to changes in the social environment, such as advances in medical technology, a heightened awareness of people’s rights, and the diversification of values. In Japan, a wide variety of ethical issues are experienced by nurses, as shown in the 35 items listed as ethical issues experienced by clinical nurses [1] by Ogawa et al. Above all, the debate about the pros and cons of physical restraint and artificial nutrition via gastrostomy is a stumbling block for nurses in developed countries because they are either aiming to protect the medical judgment and dignity of the subject or trying to be advocates [2–5]. There are also common and everyday ethical issues related to nursing [6].

However, in the busy daily work of nursing, there is little room to fully consider ethical issues and improve one’s practical skills; many nurses are worried about ‘remorse for patient involvement’, ‘avoidance of problem involvement’, and ‘negative feelings about people who have conflicting ideas’ in their current situation [7]. There are also reports that clinical nurses with high levels of ‘moral sensitivity’ suffer from greater fatigue [8], while nurses with low levels of ethical sensitivity have lower burnout levels than nurses with high levels of ethical sensitivity [9].

There are challenges related to an ethical education that enhances individual ethical practices and fosters an organizational culture that can actively tackle ethical issues and support staff who are suffering, because the ethical practices of nurses currently affect the quality of care [10]. It has also been shown that improving the ethical environment is related to the job satisfaction of nurses and their effective collaboration with physicians [11, 12]. From the perspective of team-oriented medical care, it is also necessary to have the ability to actively comment from a nursing standpoint and to discuss ethical issues in the field of multidisciplinary collaboration. While it is evident that strengthening ethical practices is becoming more important, a concrete practical list of what is ethically competent and practiced has not been clear until now.

The World Health Organization (WHO) Global Competency Model includes definitions and effective behaviors and emphasizes the relationship of these behaviors to professional competencies in ethics as ‘behaviors consistently in accordance with clear personal ethics and values’. [13] From the perspective of the United States and WHO European countries, the term ‘competence’ relates to a combination of knowledge, skills, attitudes, and values. Competency is therefore a combination of attributes underlying some aspect of successful professional performance [14].

Lechasseur et al. showed that the most frequently used terms with regard to ethical competence in nursing are ethical sensitivity, ethical knowledge, ethical reflection, ethical decision-making, ethical action, and ethical behavior [15]. Kulju et al. defined the concept of ethical competence in the context of health care settings as character strength, ethical awareness, moral judgment skills and the willingness to do good [16]. Maluwa also showed a high degree of abstraction of moral competence [17] but did not create a list of specific ways that nurses should practice. In nursing practice, the process of thinking and the actions that accompany this process are emphasized. According to Gallagher and Jormsri et al., moral or ethical competence in nursing practice includes the perception or recognition of ethical situations and the judgment of whether an action is in the best interest of the people who require nursing care [18, 19]. These studies, however, do not contain a concrete practical list of ethical caring competencies. Although there are tools for measuring ethical competence in a particular area of nursing [20–22], these tools are difficult to generalize widely because they are specialized in a specific area.

Katayama et al. extracted the Ethical Caring Competencies List (ECCL) in nursing, which is based on issues such as concreteness and versatility and includes the aspects of thinking and behavior shown above [23]. Furthermore, based on the ECCL, Katayama et al. prepared a draft of the Ethical Caring Competency Scale (ECCS) [24].

### Conceptual framework of the ECCS

Here, we first give an overview of the philosophical foundations of the moral competence scales that have been developed and used thus far and then explain the conceptual framework of the ECCS.

Kohlberg defined moral judgment competence as ‘the capacity to make decisions and judgments which are moral (i.e., based on internal principles) and to act in accordance with such judgments’. [25] Colby *et al*. developed the Moral Judgment Interview (MJI) [26], and Lind developed the Moral Judgment Test [27] to measure moral judgment/reasoning based on Kohlberg’s theory of moral development. Additionally, Rest developed the Defining Issues Test (DIT) [28] based on Kohlberg’s theory. Rest indicated that moral behavior is formulated by four psychological components, namely, ‘moral sensitivity’, ‘moral judgment’, ‘moral motivation’, and ‘moral character’ [28]. The DIT has been used in several studies in more than 40 countries, especially in the 1970s and 1980s [28]. The MJI and the DIT have been mainly utilized in nursing studies [29, 30].

However, the results of nursing studies that have used these tools have indicated that nurses and nursing students have consistently lower than expected levels of moral reasoning [31]. Furthermore, the results regarding the relationships among the variables of moral judgment, education level, and ethical behavior for nurses or nursing students are unclear [29, 30, 32, 33]. In response to these results, many nursing researchers have criticized Kohlberg’s theory for focusing on a justice-oriented conception of morality, which is used more frequently by men [29, 30, 32, 34]. A justice-oriented conception of morality is a theory that judges an act based on whether or not the act is consistent with a specific ‘ethical obligation’. These authors have also suggested that Gilligan’s sex-related theory [35] should be considered; consequently, the use of MJI and DIT has declined in nursing research.

Koskenvuori et al. introduced the four most recent scales in a scoping review of health care professionals’ ethical competence [36]. These are the Moral Competence Scale for Home Care Nurses (MCSHCN) [20], the Moral Competence Questionnaire for Public Health Nurses (MCQ-PHN) [21], the Moral Competence Scale (MCS) [37], and the Moral Skills Inventory (MSI) [38]. In three of these scales (the MCSHCN, the MCQ-PHN, and the MSI), the structure of the instrument follows the four-component model for determining moral behavior described by Rest [28]. The other scale does not follow any previous model. These facts mean that, despite the criticisms of Kohlberg’s theory and suggestions to consider Gilligan’s sex-related theory, no such measuring tool has yet been developed.

Gilligan’s theory is also known as ‘ethics of care’ or ‘care ethics’. Ethics of care is the opposite of a justice-oriented conception of morality. Ethics of care focuses on the responsibilities of multiple people in disagreement and their networks and explains these responsibilities in a contextual and narrative way of thinking. Recently, Toronto has shown four ethical elements of caring, namely, attentiveness, responsibility, competence, and responsiveness [39]. Although the ECCL was derived from a qualitative and descriptive analysis of the data obtained from interviews, it has been shown to fit very well into these four ethical elements. Therefore, the ECCL is currently viewed as a rare list of competencies for health care professionals with a theoretical background in ethics of care.

### Preparation of an ECCS draft

In assessing nursing competence, it is important to consider the context within which it is to be used [19, 40]. This is especially important for moral competence assessment, as such assessment should reflect the actual behavior of nurses in ethical situations [34]. Additionally, the nursing competence required for effective performance in nursing practice has been mainly defined as an integrated set of knowledge, skills, traits, and attitudes [41–45]. Even though Fry and Johnstone [46] acknowledged the importance of ethical practice in producing quality care, and moral competence has been described as one of the professional components in nursing [47], a highly applicable scale to measure nurses’ ethical competence has not been developed.

The draft ECCS, which is based on the concept of caring ethics, was created by Katayama et al. through a qualitative and descriptive study and has confirmed content validity and criterion validity [23, 24]. The data used for qualitative descriptive analysis were obtained from interviews held with 15 nurses (mean age, 41.5 ± 5.2 years; mean work experience as a nurse, 19.9 ± 5.2 years). The draft ECCS consists of the four core competencies and a total of 22 items of competency. The consistency between Toronto’s theory and the ECCS has been confirmed as follows. Toronto’s first element, namely, ‘attentiveness’, is included in ECCS items such as ‘Estimates the patient’s subjective distress from physical assessment’ and ‘Feeling conflicted and uncomfortable about situations where good care is not being provided’. The second element, namely, ‘responsibility’, is included in ECCS items such as ‘Expresses values about good care in individual cases’ and ‘Explores diverse values and awareness without sticking to own values’. The third element, namely, ‘competence’, is included in ECCS items such as ‘Introduces evidence into practice with appropriate procedures’ and ‘Disseminates and raises issues without ignoring the challenges of performing good care’. The fourth element, namely, ‘responsiveness’, is exactly the same as the ECCS item of ‘The evaluation of care is based on reactions such as the words and behavior of the patient and/or their family’. The draft ECCS can be used as an action guideline for ethical care. Moreover, the draft ECCS can be used in the creation, evaluation, and operation of an in-service education program for each difficulty level of evidence-based nursing ethics after statistical verification.

Katayama et al. also suggested that it is necessary to select the 22 competency related items by difficulty level according to the proficiency level of nurses, referring to the Dreyfus model [48]. This is because nursing skills change qualitatively according to experience and proficiency, as seen in the application of Benner’s Dreyfus model of the acquisition of nursing skills [49]. The ECCS can be used as a behavioral guideline for ethical care. In addition, the draft ECCS will be available for the creation, evaluation, and operation of evidence-based nursing ethics nurse education programs after statistical validation.

### Problem, research goals, and aim

Little has been written regarding establishing a basis for an instrument to evaluate health professionals’ concrete ethical caring competence. If a concrete ethical competence list could be developed, such a list could guide educators, as well as managers, in health care in supporting the development of ethical conduct in health care, which could lead to the possible assessment of ethical competence. Although the draft ECCS has confirmed content validity and criterion validity, its reliability and validity as an evaluation scale for each difficulty level have not yet been confirmed. In addition, statistical verification is required to examine whether the conditions of the Rubric Scale of Ethical Caring Competency (RECC) are satisfied.

Therefore, the research goals under consideration in this study were as follows:

1. To conduct the statistical verification of the reliability and validity of the ECCS as a scale and determine whether differences in ethical caring competency can be measured; and
2. To determine the possible use of the ECCS in a rubric format.

The aim of this study was to verify the reliability and validity of the difficulty setting of the items found in the draft ECCS and to obtain suggestions for the use of the scale as a RECC.

## Methods

### Study design

This cross-sectional instrumental psychometric study determined the reliability and validity of the ECCS and used an anonymously self-rated questionnaire.

### Participants

The participants consisted of a total of 1157 nurses working in two public hospitals, specifically, 486 nurses in August 2018 and 489 nurses in July 2019 from Hospital A and 182 nurses in September 2020 from Hospital B. All the participants were regularly employed nursing staff who worked in the ward at the target hospitals and were not on leave. Hospital A was investigated in two different years, but the participants were treated as different groups because many staff members changed over time. The authors recruited as many participants as possible. The reason was that, in general, 3-10 times as many participants as measurement items were needed, and Gorsuch has suggested that ‘the minimum number is 100 and the maximum is as many as possible’. [50] For the test-retest, we invited 10 nurses for whom no personnel changes were planned at that time from one specific department of Hospital An in 2018.

### Procedure for data collection

Research collaborators at Hospitals A and B collected completed questionnaires that had been sealed by the participants and delivered them directly to the researchers unopened.

### Instruments

The questionnaire consisted of the following instruments.

#### Demographics

Demographics included age, gender, years of experience as nursing staff, highest educational background, and questions about the participant’s study experience of nursing/medical ethics and efforts to prevent physical restraint use.

#### The draft ECCS (22 items)

The draft ECCS, which is based on the concept of caring ethics, was created by Katayama et al. through a qualitative and descriptive study and has confirmed content validity and criterion validity [23, 24]. The draft ECCS consists of the following four core competencies and a total of 22 competencies attached to them: ‘Expressing the sensitivity and value of good care’, ‘Acting while thinking about how to provide better care’, ‘Creating indirect effects to provide better care’, and ‘Acting to learn what better care is’. The 22 concrete items are rated on a 5-grade Likert scale (ranging from 1 to 5, where 1 = not at all, 2 = a little, 3 = neither, 4 = quite a lot, 5 = very much), and higher scores indicate a higher level of competency.

#### Work Motivation Measurement Scale for Nurses (15 items)

The Work Motivation Measurement Scale for Nurses was developed by Sano et al., who also verified the scale’s reliability and validity [51]. The Work Motivation Measurement Scale for nurses consists of 15 items in two subscales. A reliability analysis of the two subscales gave Cronbach’s alpha coefficients of .93 and .86, respectively. Fifteen items are rated on a 5-grade Likert scale (ranging from 1 to 5, where 1 = strongly disagree, 2 = disagree, 3 = neither, 4 = agree, 5 = strongly agree), and higher scores indicate higher work motivation.

Previous studies have hypothesized that the ECCS and work motivation show a positive correlation because of the positive correlation between ethical behavior and work motivation [24, 52]. Therefore, in this study, the Work Motivation Measurement Scale for Nurses was used to confirm the conceptual validity of the ECCS by supporting the hypothesis.

### Statistical analysis

The analysis was carried out according to the Consensus-based Standards for the Selection of Health Measurement Instruments (COSMIN) Risk of Bias checklist [53] and the Health Measurement Scale: A Practical guide to Their Development and Use, Fifth Edition [54]. SPSS for Windows ver. 26 and Amos for Windows ver. 26 were used for the analysis. For all statistical analyses, a two-sided *p* < .05 was considered significant.

#### Sample size

An exploratory factor analysis was performed, and a goodness-of-fit test was performed by the Kaiser–Meyer–Olkin measure of sampling adequacy [55] and Bartlett’s test of sphericity.

#### Reliability and validity of ECCS as a measure

1. Item selection

First, item analysis was carried out for the sealing effect, floor effect, item-total correlation (≥ 0.3), and Cronbach’s α coefficient when the item was eliminated. Next, an exploratory factor analysis was performed (principal factor method, promax rotation). Three criteria were used in selecting the factors and items within a factor: (a) an item-factor loading ≥0.4, (b) a Cronbach’s alpha ≥.70, and (c) the possibility of a factor interpretation. The number of factors was fixed at 4, which is the number of core competencies.

2) Reliability

Reliability was verified by the test-retest method, which confirms reproducibility and the Cronbach’s α value of the four core competencies.

3) Validity

The construct validity was verified by fit of the model based on a confirmatory factor analysis of the ECCS (GFI < .84, GFI > AGFI, CFI < .85, RMSEA >.10) [53] and the Pearson’s correlation coefficient of the four core competencies and work motivation. Construct validity was also verified by differences in the total score of competencies according to the presence or absence of effort to reduce physical restraint, the presence or absence of study experience of nursing/clinical ethics, and years of experience.

#### Validity of selecting ECCS items according to the proficiency level of nurses

Three difficulty levels were constructed by relative comparison of average scores of the 22 items in the four core competencies. The average score was classified based on the criteria of < 3.3 for level A, 3.3-3.5 for level B, and > 3.5 for level C after discussions by the researchers. Within the four core competencies, the mean score was analyzed by unpaired *t test* or one-way analysis of variance. In addition, to verify the validity of the three levels, we analyzed the difference in competency scores in three groups of participants based on years of nursing experience, i.e., an expert group (*n* = 168), a proficient group (*n* = 407), and a competent group (*n* = 387). For convenience, this grouping was based on Benner’s theory [49] with 0-6 years of experience defined as a ‘competent group’, 7-24 years of experience defined as a ‘proficient group’, and 25 years or more of experience defined as an ‘expert group’.

### Ethical considerations

This study was approved by the Clinical Research Ethics Committee of Hamamatsu University School of Medicine (Decision no. 18-267). Nurses were informed that participation in this study was voluntary and that refusing to participate would not incur any negative consequences. They were also told that their answers would be anonymous and confidential and would not be used for any purpose other than this study. Consent was confirmed by filling in the consent confirmation column for participation in the questionnaire and posting it in the collection box. The questionnaires were returned in a sealed envelope to a collection box located at each of the participating hospitals. The participants had the freedom to not answer questions and were allowed to return incomplete or blank questionnaires.

## Results

### Participants

Of the total 1157 people who were sent the questionnaire, 962 provided valid responses (valid response rate = 83.1%). Of these, 98.1% were regular nurses, and approximately half of them had less than 10 years of experience as a nurse. Regarding the highest academic credentials earned, 68.2% had attended a 3-year nursing college (Table 1). The Kaiser–Meyer–Olkin measure of sampling adequacy was .931, and Bartlett’s test of sphericity was <.0001.

**Table 1:** Characteristics of the participants

		*n* = 962
Variables	n	%
Facilities		
Hospital A at baseline	452	47.0
Hospital A after 1 year	425	44.2
Hospital B	85	8.8
Nursing experience (years)		
0-2	180	18.7
3-9	291	30.2
10-19	220	22.9
20-29	189	19.6
≥ 30	71	7.4
Unknown	11	1.1
Sex		
Female	886	92.1
Male	67	7.1
Unknown	9	0.8
Educational background		
Nursing school (2-year course)	90	9.4
Nursing school (3-year course)	656	68.2
Junior college graduate	85	8.8
College graduate	110	11.4
Graduate school	11	1.2
Unknown/other	10	1.0
Study experience in nursing/medical ethics		
Yes	567	58.9
No	389	40.4
Unknown	6	0.6
Effort to prevent physical restraint		
Yes	656	68.2
No	283	29.4
Unknown	23	2.4

### Reliability and validity of ECCS as a measure

1. Item selection

None of the 22 ECCS items showed a ceiling effect or floor effect. The item-total correlation ranged from .529 to .736. The Cronbach’s α coefficient when the item was eliminated ranged from .926 to .930 (Table 2). In an exploratory factor analysis (principal factor method, promax rotation), the highest value of an item factor loading was ≥0.4 for all items, and the number of factors also converged well at four. The Cronbach’s α coefficients of the four factors all showed ≥.70. Therefore, no items were excluded.

2) Reliability

**Table 2:** Fundamental statistics of the 22 competencies and degree of difficulty composition from level A to C

													n = 962
Four core competencies and 22 competencies	Min	Max	Mean	SD	Mean + SD	Mean-SD	Item-total correlation	α when the item was eliminated	Item factor loading	Difference in difficulty level			
										Level	Mean	SD	*p*
I. Expressing the sensitivity and value of good care (α = .717)													
1. Expressing values about good care in individual cases	1	5	3.01	.69	3.70	2.32	.529^**^	.930	.599	A	3.14	.57	< .001
2. Having a bird’s-eye view of the pros and cons of caring based on laws, rules and social trends	1	5	3.27	.66	3.93	2.61	.580^**^	.929	.723				
3. Exploring diverse values and awareness without sticking to own values	1	5	3.31	.71	4.02	2.60	.543^**^	.930	.676	B	3.36	.59	
4. Feeling conflicted and uncomfortable about situations where good care is not being provided	1	5	3.41	.76	4.17	2.65	.557^**^	.930	.432				
II. Acting while thinking about how to provide better care (α = .873)													
1. Evaluation of care is based on reactions such as the words and behavior of the patient and (or) their family	1	5	3.30	.73	4.03	2.57	.521^**^	.930	.402	A	3.30	.73	< .001 in all pairs
2. Practice good care patiently without giving up	1	5	3.30	.71	4.01	2.59	.636^**^	.928	.412	B	3.40	.58	
3. Introduce evidence into practice with appropriate procedures	1	5	3.35	.71	4.06	2.64	.634^**^	.928	.413				
4. Support the patient and (or) their family to gain essential awareness	1	5	3.42	.77	4.19	2.65	.707^**^	.927	.806				
5. Support decision making in the way and at the pace the patient and (or) their family wants and create a care plan together	1	5	3.53	.80	4.33	2.73	.676^**^	.927	.805				
6. Perspective taking of the patient and (or) their family’s experience by observing behavior and integrating multifaceted information	1	5	3.53	.68	4.21	2.85	.616^**^	.929	.437	C	3.60	.55	
7. Estimate patient’s subjective distress from physical assessment	1	5	3.54	.69	4.23	2.85	.547^**^	.930	.774				
8. Create relationships so the patient and (or) their family can talk about important things	1	5	3.74	.75	4.49	2.99	.629^**^	.928	.768				
9. Accept the patient and (or) their family facing the rigors of reality	1	5	3.58	.75	4.33	2.83	.670^**^	.928	.826				
III. Creating indirect effects to provide better care (α = .890)													
1. Flexibly create contextual care teams based on professionalism and expertise	1	5	3.04	.87	3.91	2.17	.715^**^	.927	.957	A	3.14	.73	< .001 in all pairs
2. Create and modify systems for good care, such as conferences	1	5	3.18	.83	4.01	2.35	.688^**^	.927	.934				
3. Understand and discuss the conflicts of team members based on multidisciplinary collaboration	1	5	3.19	.83	4.02	2.36	.664^**^	.928	.653				
4. Disseminate and raise issues without ignoring the challenges of performing good care	1	5	3.33	.80	4.13	2.53	.736^**^	.926	.662	B	3.33	.80	
5. Understand and discuss conflicts between colleagues or department members	1	5	3.46	.76	4.22	2.70	.664^**^	.928	.933	C	3.50	.69	
6. Exchange opinions and participate in conferences in order to carry out good care	1	5	3.52	.78	4.30	2.74	.702^**^	.927	.450				
IV. Acting to learn what better care is (α = .793)													
1. Discover and disseminate ethical research issues from practice	1	5	2.83	.88	3.71	1.95	.645^**^	.928	.674	A	2.83	.88	< .001 in all pairs
2. Learn about good care from reflection and insights based on the experience case	1	5	3.29	.77	4.06	2.52	.700^**^	.927	.577	B	3.29	.77	
3. Gain awareness about good care practices at learning opportunities	1	5	3.35	.75	4.10	2.60	.681^**^	.927	.559	C	3.35	.75	

Level A is the most difficult level

**p < .001

α = Cronbach’s α coefficient

*SD* standard deviation

The Cronbach’s α coefficient of the four core competencies ranged from .717 to .890. We also confirmed *r* = .900 (*p* < .001) by the test-retest method.

3) Validity
4) Structural validity

**Fig. 1:**
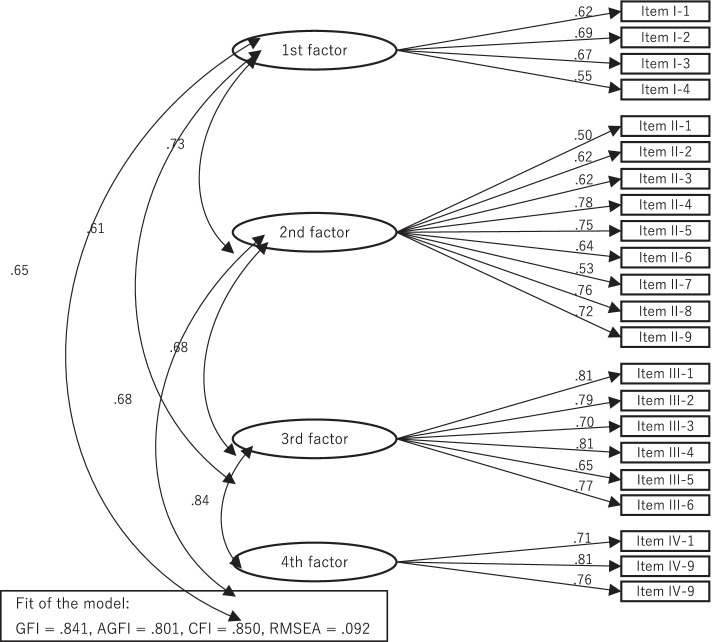
Confirmatory factor analysis of the ECCS

As a result of verifying the structure of the four factors by confirmatory factor analysis, the goodness-of-fit index (GFI) was .841, the adjusted goodness-of-fit index (AGFI) was .801, the comparative fit index (CFI) was .850, and the root mean square error of approximation (RMSEA) was .092 (Fig. 1).

(2) Construct validity

The competency score for ‘yes’ respondents regarding making an effort to reduce the use of physical restraint was 74.5 points, which was significantly higher than the 71.3 points scored for ‘no’ respondents (*p* < .001). The competency score of the respondents who had participated in training on nursing/medical ethics was 75.0 points, which was significantly higher than the 71.9 points scored for the respondents who answered ‘no’ (*p* < .001). Those with a longer duration of experience as a nurse tended to have a higher competency score than did those with a shorter duration (*p* < .001 for multiple combinations) (Table 3).

**Table 3:**
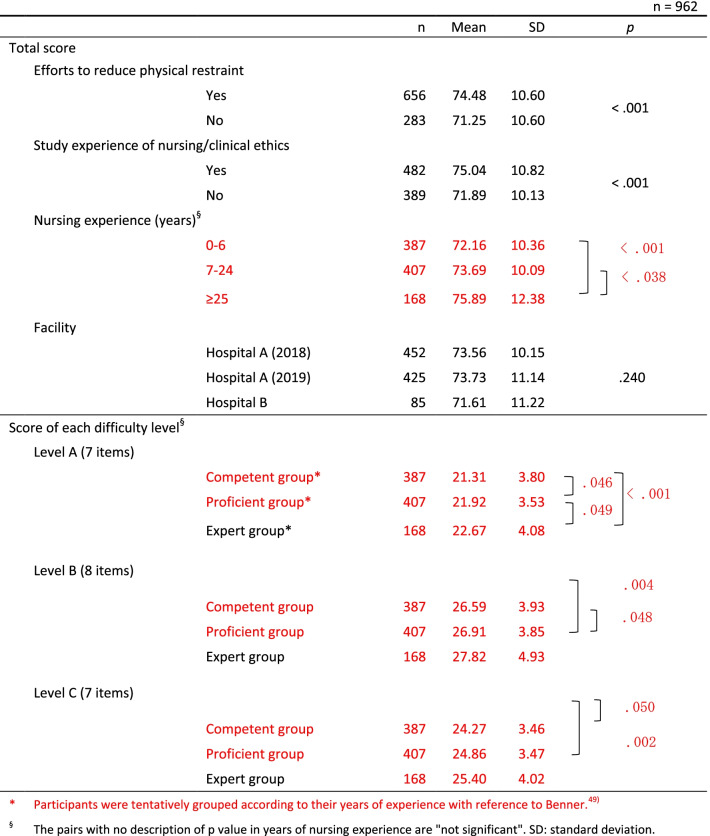
Differences between groups in the ECCS for participant’s variables.

Pearson’s correlation coefficient *r* between the score of the four core competencies and the score of work motivation ranged from .232 to .367 (in either case *p* < .001). In addition, the scores of the four core competencies had a higher correlation coefficient with ‘future work motivation’ than with ‘current work motivation’ (Table 4).

**Table 4:** Correlation between scores of the ECCS and work motivation

		*n* = 962
	Work motivation	
	Current	Future
1st factor	.257^**^	.329^**^
2nd factor	.311^**^	.367^**^
3rd factor	.237^**^	.264^**^
4th factor	.232^**^	.310^**^
Pearson’s correlation coefficient		

**p < .001

### Validity of selecting ECCS items according to the proficiency level of nurses

According to the relative ranking of the average scores of each of the 22 items, three difficulty levels ranging from level A to C were composed within four core competencies (Table 2). The difference in the total score of the items at the three difficulty levels in the four core competencies was significantly different in all pairs (A > B > C) (*p* < .001, in all pairs) (Table 2). In addition, the competency scores according to the levels of the expert group (*n* = 168), the proficient group (*n* = 407), and the competent group *(n* = 387) showed significant differences in most pairs (Table 3).

## Discussion

### Participants

The number of participants was 962, which was at least ten times larger than the 22 items included in the ECCS and ≥ 100; therefore, the sample size was suitable for factor analysis [54]. The Kaiser–Meyer–Olkin measure of sampling adequacy [55] and Bartlett’s test of sphericity showed goodness of fit.

Nursing manager competencies are believed to increase with years of experience [24, 56]. It has also been shown that ethical sensitivity, which is defined as the ability to recognize perceived ethical discomfort as a ‘problem’, is also associated with increased nursing experience [24, 57, 58]. Based on this, it can be estimated that the competencies evaluated by the ECCS may differ qualitatively and quantitatively depending on the number of years of experience as a nurse. Therefore, it is considered that approximately half of the participants had less than 10 years of experience as a nurse, which was suitable for verifying the difficulty level of the ECCS.

Furthermore, although the new fiscal year in Japan starts in April, this study was conducted in August, November, and June; since these are times when workloads are relatively stable, the measurement time chosen was most likely appropriate.

### Reliability and validity of ECCS as a measure

1. Item selection

Since neither a ceiling effect nor a floor effect were shown, it was judged that there were no items with biased scores. The item-total correlation showed that all items exceeded .3. Five of these items exceeded .7 (II-4, III-1, III-4, III-6, IV-2), but all are necessary and indispensable items for evaluating ethical caring competency [24]. In addition, since the number of items was 12 or more, the sample size exceeded 300, and the Cronbach’s α coefficient when an item was eliminated exceeded .9 for all the items, it was confirmed that the reliability of the items was high [53]. All the items reached the criteria for an item-factor loading ≥0.4, a Cronbach’s alpha ≥.70 in the exploratory factor analysis (principal factor method, promax rotation), and had the possibility of a factor interpretation. Therefore, no items were excluded.

2) Reliability

The *r* in the test-retest reliability was as high as 0.9, thereby confirming the reproducibility of the ECCS. The Cronbach’s α coefficients of the four core competencies were also above the criteria values. Based on these results, the reliability of the ECCS was confirmed.

3) Validity
4) Structural validity

Structural validity was confirmed to be almost suitable by confirmatory factor analysis (GFI < .84, GFI > AGFI, CFI < .85, RMSEA >.10) [53].

(2) Construct validity

The ECCS score was significantly higher for those who were working to reduce the use of physical restraint, those who had learning experience in nursing/medical ethics, and those who had many years of experience. Routine ethical care practices and learning are thought to increase one’s level of ethical sensitivity [59]. Competencies increase with years of experience [56], and years of experience affect the ethical sensitivity of nurses [57, 58]. Based on this, it is reasonable that the competency evaluated by the ECCS also showed a high score for those who are making an effort to reduce their use of physical restraint, those who have learning experience in nursing/medical ethics, and those who have many years of experience. Therefore, the validity of the ECCS can be verified based on the characteristics of the participants.

In addition, the four core competencies were significantly associated with current and future work motivation. This outcome is consistent with the competency rudder concept, which shows that proficiency that continues to mature while reflecting on experience affects work motivation and job satisfaction [51]. Therefore, it was confirmed that the ECCS can appropriately evaluate the competencies of ethical care in nursing practice. Ethical caring competency in the clinical nursing setting is defined as follows: nurses who have learned the principle of ethics discuss that what is best for the patient is being hindered in daily care. It is also a competence that can be displayed through a series of actions that work indirectly and directly to promote consensus building for what is best for the patient and continue to mature when one reflects on one’s experience [24]. Convergent validity was confirmed by testing the hypothesis that there is a positive correlation between the four core competencies and work motivation.

### Possibility of use as an evaluation form in rubric format

The total score for each of the three difficulty levels for each of the four core competencies showed a significant difference. This result indicates that the difficulty level of the evaluation sheet items set in this study was statistically supported. This outcome not only verified the reliability and validity of the ECCS as a general evaluation scale but also suggested that the scale could be used as a rubric format evaluation sheet.

A rubric is a tabular tool that sets up several evaluation viewpoints for the evaluation of a certain task and shows the achievement level for each stage in short sentences. Rubrics have been used for self-evaluations and program evaluations in recent years [60]. Rubrics have the property of concretely exemplifying and expressing the content when considering whether the required quality is achieved [61]; therefore, it can be said that it is a suitable format for the evaluation of good care targeted by the ECCS.

## Limitations

The main goal of the study was to develop a rubric scale of ethical caring competency. It is necessary to arrange the format, determine the rules of the scoring method, and implement the scale. Due to the limited scope of this study, there is a limit to its widespread generalization. Therefore, it is necessary to analyze data drawn from a wider variety of participants, with the aim of making the scale adaptable to multidisciplinary health care providers, standardizing the ECCS, and implementing it extensively.

## Conclusion

The reliability and validity of the ECCS as a scale were statistically verified, and we were able to obtain suggestions for its application for use as an evaluation form in rubric format.

## Data Availability

The datasets generated and/or analyzed during the current study are not publicly available due to still being under analysis but are available from the corresponding author upon reasonable request.

## Abbreviations

ECCS: Ethical Caring Competency Scale
